# Health-related quality of life and needs of care and support of adult Tanzanians with cancer: a mixed-methods study

**DOI:** 10.1186/1477-7525-10-133

**Published:** 2012-11-02

**Authors:** Golden M Masika, Lena Wettergren, Thecla W Kohi, Louise von Essen

**Affiliations:** 1 School of Nursing, College of Health Sciences, The University of Dodoma, P.O. Box 259, Dodoma, Tanzania; 2 Department of Public Health and Caring Sciences, Psychosocial Oncology and Supportive Care, Uppsala University, Box 564, Uppsala 751 22, Sweden; 3 Department of Neurobiology, Care Sciences and Society, Division of Nursing, Karolinska Institutet, 23 300, Huddinge 141 83, Sweden; 4 School of Nursing, Muhimbili University of Health and Allied Sciences, P.O. Box 65004, Dar es Salaam, Tanzania

**Keywords:** Cancer, Care, Health-related quality of life, Support, Tanzania

## Abstract

**Background:**

Cancer is among the three leading causes of death in low income countries and the highest increase with regard to incidence figures for cancer diseases are found in these countries. This is the first report of the health-related quality of life (HRQOL) and needs of care and support of adult Tanzanians with cancer.

**Methods:**

A mixed-methods design was used. The study was conducted at Ocean Road Cancer Institute (ORCI) in Dar es Salaam, Tanzania. One hundred and one patients with a variety of cancer diagnoses treated and cared for at ORCI answered the Kiswahili version of the EORTC QLQ-C30 investigating HRQOL. Thirty-two of the patients participated in focus group interviews discussing needs of care and support. Data from focus group interviews were analyzed with content analysis.

**Results:**

The findings show that the patients, both women and men, report a low quality of life, especially with regard to physical, role, and social function and a high level of symptoms and problems especially with financial difficulties and pain. Financial difficulties are reported to a remarkably high extent by both women and men. The patients, both women and men report least problems with emotional function. A content analysis of the interview data revealed needs of food and water, hygienic needs, emotional needs, spiritual needs, financial needs, and needs of closeness to cancer care and treatment services.

**Conclusion:**

The high score for pain points out that ORCI is facing severe challenges regarding care and treatment. However, when considering this finding it should be noted that the pain subscale of the Kiswahili version of the EORTC QLQ-C30 did not reach acceptable internal consistency and showed less than satisfactory convergent validity. This also applies to the subscales cognitive function and global health/quality of life. Attention should be drawn to meet the identified needs of Tanzanian cancer patients while hospitalized but also when at home. Increased accessibility of mosquito nets, pads, and pain-killers would help to fulfil some needs.

## Background

Cancer is among the three leading causes of death in developing countries and the highest increase with regard to incidence figures for cancer diseases are found in these countries. Once thought of as a ‘rich world’ disease, cancer is a looming public health catastrophe across Africa [1]. People lack access to information on how to identify early signs of different cancers. Those who seek treatment typically have few options. Medication is expensive [2]. Facilities are few and overcrowded. Compounding the challenge are the many stigmas attached to the disease.

The diagnosis and treatment of cancer often has an impact on health-related quality of life (HRQOL) and cause multiple concerns and needs of care and support. HRQOL is typically measured with standardized instruments such as the EORTC QLQ-C30 [3]. To the best of our knowledge HRQOL of Tanzanian cancer patients has not been reported. However issues related to HRQOL have been investigated among Tanzanians with cancer in some studies. The aims of one of these were to identify symptoms experienced by Tanzanian females with cervix cancer and causes of late presentation [4]. It was shown that more than 90% reported late with advanced disease. The aims of another study [5] were to explore physicians’ decisions concerning disclosure of diagnostic, prognostic, and referral information and the effect of cultural, economic, and social factors on how this information is handled. The results show that Tanzanian physicians often withhold diagnostic and prognostic information from patients [5]. Cultural arguments, issues of treatment availability, and patient poverty influenced the disclosure practices [5]. The findings are supported by results from a study by Mkiramweni and co-workers [6] regarding referral delay for persons from the rural part of Tanzania and its influence on the management of cancer demonstrating that a fourth of the participants were not informed that they had cancer when arriving at the health care facility. End of life care has been investigated in some studies from Sub-Saharan Africa including Tanzania [7,8].

In Tanzania the health care is delivered at dispensaries which serve at the village level, health centres which serve at the ward level, and hospitals which serve at the district level. Additionally there are hospitals in all regions of the Tanzania mainland and a consultant hospital in each of the four country zones. Approximately 2,500 cancer patients are yearly attended to at Ocean Road Cancer Institute (ORCI) in Dar es Salaam, the only public hospital in Tanzania providing a comprehensive cancer treatment [9]. Most patients arrive at ORCI after their disease is well into stage four, when the cancer has spread to other organs [9]. Early signs are often missed by local village health providers, who can make incorrect diagnoses and are usually not aware about the importance of informing about warning signs. Breast cancer and Kaposis sarcoma account for 33% and 10% respectively of all cancers of men and women seen at ORCI [9]. The International Atomic Energy Agency’s (IAEA) Program of Action for Cancer Therapy (PACT) and the World Health Organization (WHO) support the national authorities in implementing the Tanzanian National Cancer Strategy and Action Plan [10]. The work focused on radiotherapy and palliation, cancer surveillance, prevention, and early detection as well as strengthening civil society and community support for cancer control [11]. In addition there have been and are a number of collaborations aiming at strengthening the capacity of ORCI. However, to the best of our knowledge, no program, collaboration or study has addressed the needs of care and support and/or the HRQOL of Tanzanian cancer patients, from the patients’ perspective. We therefore, in this study, set out to explore the HRQOL as well as needs of care and support of Tanzanian cancer patients admitted at ORCI in Dar es Salaam.

## Methods

### Setting

The study has a mixed-methods design including quantitative and qualitative methods. The study was conducted at ORCI in Dar es Salaam, Tanzania mandated to provide medical care to in- and outpatients with cancer; stimulate and promote programs of education on health, particularly on cancer; conduct training programs; provide consultant services, and perform research activities to provide a better cancer control in Tanzania [9]. The hospital has two wards for females, one ward for men, and one ward for children with a total capacity of 125 beds. The bed occupancy averages to about 130% per day, hundreds of people are daily given access at ORCI to radiotherapy, nuclear medicine, and/or chemotherapy to fight cancer.

### Sample

Adult cancer patients, native speakers of Kiswahili, treated and cared for at ORCI, in a mental and physical condition to permit/manage participation (judged by the nurse in charge at the respective ward), were, during a week in August 2008, asked to answer the Kiswahili version of the original EORTC QLQ-C30 [3].

During the study week 186 patients were hospitalized at ORCI. Of these 37 were children, 41 did not meet the inclusion criteria, and 7 did not accept participation. The sample includes 101 patients who came from regions including Lindi, Mara, Mbeya, Mtwara, Mwanza, and Rukwa. Information about their working and social situation, diagnoses, and treatments is presented in Table 1 and 2 respectively. Most participants were peasants, married, and responsible for children. Forty-three percent of the women were widows or had separated from their husbands. Most women were diagnosed with cervical or breast cancer.

**Table 1:** Socio-demographic characteristics for women (n=81) and men (n=20)

	**Women**	**Men**
Working situation		
Peasants	61	11
Unemployed^a^	8	3
Professionals/Employees of lower level^b^	6	3
Small scale business^c^	6	3
Marital status		
Married	41	12
Widowed	23	3
Divorced	12	1
Unmarried	5	4
Responsible for children	72	16
Household members (besides participant) (range)	5 (0–14)	5 (2–12)

^a^housewives, house girls, students, retired officers; ^b^police officers, secretaries, hotel servers, drivers, health attendants, primary school teachers; ^c^carpenters, masonries, street vendors, electricians, tailors, other small scale business.

**Table 2:** Diagnosis and treatment characteristics for women (n=81) and men (n=20)

	**Women**	**Men**
Diagnosis		
Ca breast	15	0
Ca bladder	0	1
Ca cervical	46	0
Ca eye	3	2
Ca groin	1	0
Ca kidney	1	0
Ca larynx	0	1
Ca oesophagus	1	3
Ca prostate	0	1
Ca rectum, and colon	2	3
Ca skin and eyes	2	1
Ca tongue	0	1
Hodgkin	1	0
Kaposis sarcoma	2	3
Leukemia and lymphoma	5	3
Liposarcoma	1	0
Nasalpharyngeal carcinoma	1	0
Squamous cell carcinoma	0	1
Treatment		
Chemotherapy	17	2
Radiotherapy	47	10
Radiotherapy and chemotherapy	10	3
Radiotherapy and surgery	1	0
Surgery	2	1
Surgery and chemotherapy	3	0
Not yet started treatment	1	4

### Procedure and data collection

#### Questionnaire

As a first step of this research the EORTC QLQ-C30 [3] measuring HRQOL, originally designed and developed in English to be used among cancer patients and translated into several languages, was translated into Kiswahili by our research group. Translation was done in accordance with the procedure developed by the European Organisation for Research and Treatment of Cancer (EORTC) [12,13]. The procedure included a forward/backward translation of the EORTC QLQ-C30. The original English version was translated into Kiswahili by two independent translators who were native Kiswahili speakers with proficiency in English. A research coordinator compared the two forward translations and checked them for any discrepancies. The discrepancies between the two translations were discussed with the translators until agreement was reached on a provisional forward translation. This forward translation was then back translated into English by two independent translators who were native speakers of English with proficiency in Kiswahili. The English back translations and the original English version were compared to assure that there were no differences in the meaning of the questions in the questionnaires. The provisional Kiswahili version was pilot tested on forty-nine adult cancer patients, native speakers of Kiswahili, in a mental and physical condition to permit/manage participation, treated and cared for at ORCI. The pilot test was conducted according to the manual provided by EORTC [13]. Patients were asked to respond to the questions in the EORTC QLQ-C30 and whether any of the questions was: difficult to answer, confusing, difficult to understand, upsetting/offensive, and whether he/she would have asked the question in a different way (unpublished data). Due to the large number of patients who could not read nor write seen at ORCI the questions were read to all patients and their responses noted. Results of the translation and the pilot study were reviewed by the EORTC translation coordinator to ensure that the content and applicability was maintained, and the EORTC QLQ-C30 Kiswahili version was authorized by the EORTC Quality of Life Group. In the current study the Kiswahili version of the EORTC QLQ-C30 was used. The instrument comprises 30 questions divided into subscales measuring: global health status/quality of life, physical, emotional, social, cognitive, and role function and pain, fatigue, nausea/vomiting as well as dyspnoea, loss of appetite, insomnia, diarrhoea, constipation, and financial difficulties. It is a self-report questionnaire with a Likert format response set. The reliability and validity of the subscales in the Kiswahili version of the EORTC QLQ-C30 is satisfactory besides for cognitive function, global health/quality of life, and pain as shown in Table 3.

**Table 3:** Convergent and discriminant validity and internal consistency reliability for the Kiswahili version of the EORTC QLQ-C30

**Scales**	**No items per scale**	**Floor**/**ceiling effects(%)**	**Convergent validity(range of correlations)**	**Discriminative validity(range of correlations)**	**Scaling success(%rate)**	**Reliability(α)**
Physical function	5	0/1.0	0.41-0.62	0.05-0.62	36/40 (90%)	0.76
Role function	2	15.5/17.5	0.71	0.08-0.64	16/16 (100%)	0.83
Emotional function	4	1.9/27.2	0.53-0.77	0.09-0.50	32/32 (100%)	0.82
Cognitive function	2	1.0/15.5	0.20	0.12-0.53	3/16 (19%)	0.33
Social function	2	21.4/12.6	0.57	0.01-0.46	16/16 (100%)	0.73
Global health/QOL	2	2.9/0	0.41	0.08-0.52	10/16 (63%)	0.58
Fatigue	3	4.9/8.7	0.36-0.66	0.12-0.73	18/24 (79%)	0.71
Nausea/vomiting	2	37.9/5.8	0.63	0.00-0.31	16/16 (100%)	0.77
Pain	2	4.9/13.6	0.46	0.11-0.59	9/16 (56%)	0.63

Patients were included consecutively by the first author (GM) in cooperation with a nurse officer at ORCI. Potential participants received oral and written (if the patient could read) information in Kiswahili about the aim and procedure of the study, that participation was voluntary, and that non-participation would not affect the care and treatment. Thereafter informed consent was asked for. Patients who were unable to provide consent in written were asked to provide consent through thumb stamping. Patients were thereafter asked to respond to the questions included in the EORTC QLQ-C30. Because of the large number of patients who could not read nor write seen at ORCI the questions were read to all patients. Data were collected in the clinical facilities at ORCI by GM and the nurse officer at ORCI.

#### Focus group interviews

Participants were, in addition to the criteria presented above, asked about participation in focus group interviews according to willingness and ability to express themselves verbally as judged by a nurse in charge of the ward where the respective patient was cared for. Focus group interviews are appropriate when collecting data on unexplored areas [14,15]. Potential participants were asked about informed consent by the nurse in charge of the respective ward. The great majority of those who participated in these interviews (n=32) had answered the EORTC QLQ-C30 instrument. As breast and cervical cancers are among the most prevalent cancer diagnoses among women in Tanzania, women with such diagnosis were asked for participation in the focus group interviews. As there was a great variety of diagnoses among men admitted at ORCI during the study week, men were asked for participation in the focus group interviews regardless of diagnosis. The interviews, two with eight women each, with cervical or breast cancer and two with eight men each, with Kaposis sarcoma, oesophageal carcinoma, prostatic cancer or head and neck tumors were conducted at ORCI by the first author and a research assistant (registered nurse and doctoral student) during the study period. The patients’ needs of care and support were explored according to semi-structured interview questions about needs of care at the hospital and of help outside the hospital. The focus group interviews were led by the first author with the research assistant as an assistant observer. The research assistant took notes and observed the participants during the interviews. The first author and the research assistant have extensive experience of caring for as well as interviewing patients and their relatives. The first author started each focus group interview by introducing the aims of the interview. The interviews were audio-recorded and transcribed verbatim by a research assistant at Muhimbili University of Health and Allied Sciences, School of Nursing, Dar es Salaam, Tanzania. The transcripts were translated from Kiswahili into English by a licensed translator.

### Data analysis

The psychometric analyses of the EORTC QLQ-C30 data were analyzed with SPSS Version 20 whereas descriptive data for the EORTC QLQ-C30 were analyzed with SPSS Version 17. The Pearson correlations of an item with its own scale (corrected for overlap) and other scales were calculated to assess convergent and discriminant validity. Evidence of item convergent validity was defined as a correlation above 0.40 with its own scale. Evidence of item discriminant validity was based on a comparison of correlation of an item with its own scale and with other scales. Scaling success for any scale is defined as the number of convergent correlation coefficients significantly higher than the discriminant correlation coefficient divided by the total number of correlations. The internal consistency reliability of the instrument was assessed by the Cronbach’s alpha coefficient. According to the generally accepted standard an alpha value should not fall below 0.70 [16]. Scale scores were calculated by averaging items within scales and transforming average scores linearly. All scales range in score from 0 to 100. A high score for a functional scale represents a high/healthy level of functioning whereas a high score for a symptom scale or item represents a high level of symptomatology or problems [3]. The data are presented by means and standard deviations for the total sample and for women and men respectively. The data from the focus group interviews were analyzed by content analysis [17,18] by the first and last author. Content analysis can be used to draw conclusions about a manifest message in a communication by systematic identification of specified communication characteristics. Answers to semi-structured open-ended questions are suitable for this technique. The analysis was performed by the first and last author in the following steps: 1. The entire transcribed and translated text was read and words and sentences (recording units) that contained information relevant to the interview questions were identified. 2. Recording units were grouped into mutually exclusive categories reflecting central text messages. 3. Boundaries of each category were defined and the final descriptions of the central characteristics of each category were developed. During the analysis process, content of the categories were discussed among the first, second, and last author until agreement was reached.

### Ethical considerations

Potential participants were informed verbally and in writing (if the patient could read) in Kiswahili about the aim and procedure of the study, that participation was voluntary, that confidentiality was guaranteed, that they could withdraw from the study at any time without any consequences, and that neither participation nor non-participation would affect their treatment and care. Thereafter informed consent was asked for. Patients who were unable to write were asked to sign through thumb stamping. Special consideration was taken to patients’ dignity, integrity, and vulnerability during interviews and observations according to The Declaration of Helsinki [19]. Ethical approval was received from the Tanzanian Commission for Science and Technology, the National Institute for Medical Research in Tanzania as well as the Research Board at ORCI. Fluent speakers of Kiswahili were used to translate instruments, interview questions, answers to interview questions, and information letters. And, all contacts with patients have been taken by fluent speakers of Kiswahili.

## Results

The sample includes 101 participants from 19 to 78 years of age (X=47, SD=13), 81 of these are women (X=47, SD=14, range=19-78) and 20 are men (X=45, SD=16, range=20-72).

### Health related quality of life

The mean values and standard deviations for EORTC QLQ-C30 scales and single items for the total sample and for men and women respectively are shown in Table 4.

**Table 4:** Mean values and standard deviations for the EORTC QLQ-C30 scales and single items for the total sample (N=101), women (n=81) and men (n=20)

	**Total sample**	**Women**	**Men**
Physical function^a^	53.5 (21.8)	54.3 (21.5)	49.5 (23.3)
Role function^a^	47.8 (35.3)	49.4 (35.6)	40.4 (34.4)
Emotional function^a^	71.8 (28.5)	71.4 (29.2)	75.4 (25.1)
Social function^a^	39.0 (33.6)	41.3 (34.8)	29.8 (28.1)
Cognitive function^a^	64.0 (23.8)	65.4 (25.0)	58.8 (17.9)
Global health/QOL^a^	49.5 (22.4)	50.5 (22.5)	45.6 (22.5)
Fatigue^b^	52.9 (27.6)	52.1 (27.0)	56.4 (31.1)
Nausea and vomiting^b^	30.0 (32.2)	31.0 (32.2)	25.4 (33.5)
Pain^b^	61.7 (27.8)	60.8 (28.0)	65.8 (28.0)
Dyspnoea^b^	22.0 (33.6)	21.3 (32.4)	26.3 (39.4)
Insomnia^b^	42.7 (39.7)	40.0 (40.9)	54.4 (33.7)
Appetite loss^b^	38.5 (37.9)	39.9 (38.5)	31.6 (36.0)
Constipation^b^	40.3 (39.4)	40.0 (39.1)	40.4 (42.4)
Diarrhoea^b^	13.3 (24.2)	14.6 (25.5)	8.8 (18.7)
Financial difficulties^b^	84.3 (47.0)	84.2 (49.5)	84.2 (37.5)

^a^range 0–100, higher scores represent a high/healthy level of functioning and a high quality of life; ^b^range 0–100, higher scores represent a high level of symptoms/problems.

Social, role, and physical function are the scales given the lowest mean value by the total sample as well as by women and men respectively whereas emotional function is given the highest mean value by the total sample as well as by women and men respectively. The symptom scale rated highest by the total sample as well as by men and women respectively is the pain scale, reflecting a high level of problems with pain. The single item rated as the far most problematic by both men and women is financial difficulties. Taken together the total sample as well as men and women respectively appear to experience most problems with financial difficulties, pain, and social and role function as measured with the EORTC QLQ-C30.

### Needs of care and support

The focus group interviews provided rich data from which an analysis within and across groups was undertaken. All areas in the interview guide were more or less frequently discussed. The participants did not interact so much with each other during the interviews. The interaction was mostly between the interviewer and one participant at a time. The content analysis resulted in six categories, these are presented below.

#### Needs of food and water

Patients expressed a need of water and a variety of food. Patients were served food at ORCI, however were not satisfied with being served the same food i.e. ugali and beans every day, sometimes delayed and cold. Drinking water was not supplied for free and patients expressed a need of such water. The men, especially those with oesophageal cancer, expressed a need for special food as they could not swallow the ugali and beans provided at the hospital. They also mentioned that they spent a lot of money on bottled water.

#### Hygienic needs

Most patients came to ORCI without extra clothes thinking that they would stay only a few days at the hospital. Besides extra clothes patients, especially those with cervical cancer, needed pads to maintain hygiene and prevent smelling and infection. As pads were not available reused pieces of cloths were used in the place of pads.

Mosquito nets and bed sheets were mentioned. It was mentioned that bed sheets were not changed for days. One woman said: “*Cleanliness of the bed sheets is the cleanliness of a woman*. *If you are dirty*, *they will give out bad smell*. *But if you have many you can change but if you don*’*t have*, *you will wait until the day of changing*. *If you are dirty*, *even if they give you one sheet per day it will smell but if you have two you will change*.” The interviewer asked: *How many times do they change for you*? The patient responded: “*Aah*! *Per week they change only once*, *for a month eight is many*. *Some of us are sick*, *if you get malaria*, *you get to be bed ridden*.” (woman, group 1).

#### Emotional needs

Patients from distant regions did not receive visitors and expressed a need to see their relatives, even though pessimistic about the possibility. A woman in group 1 said: “*We need them but the possibility of coming to see us is not there*. *It is far and our relatives are living far from here*.” A man in group 4 said: “*Not everyone will hold five hundred shillings*. *It is not possible*, *people are poor*. *There was a period when I was hospitalized at Kibena hospital in Njombe*. *My son in law did not come to see me*. *When I asked him*, *he said he had no money*. *He said without money I cannot*. *Then I told him*, *I did not need money*, *when you come to see me I get comfort*. *Then I had to be mad on him*. *I asked him:* “*Are you waiting for someone to die so that you go and bury him*? *I need you to come and see me*. *It is not necessary that you look for money to come and see me*.”

Patients asked for groups of patients and other people who could visit and provide a possibility to express needs and share experiences: “*There could be an association meant for visiting patients*, *the ward executive can set a program of providing support for cancer patients*.” (woman, group 2).

Patients also expressed a need of help with taking care of their homes as there was no one to take care of the household activities they had been doing, worries that the homes might have been destroyed were expressed.

#### Spiritual needs

Patients described going to the church and the mosque at ORCI to pray and being visited by people from different church congregations. They wished more frequent visits and prayers providing them with hope. It was mentioned that this kind of visits had become less frequent during the last years.

#### Financial needs

A greater part of the discussion in all groups concerned economic challenges. A number of needs of financial support from relatives, community or government to cover for different costs were expressed. Apart from the prayers that the Christian groups brought when visiting, patients wished these groups to bring something more such as soap, clothes, or sugar. Patients needed money to cover expenses for transport from home to ORCI and back home. One patient said: “*For example the mother of Hawa*. *She came to Muhimbili* (hospital in Dar es Salaam) *with breast cancer*. *She was operated and came here*. *Here*, *she was told to go home and look for money*. *Reaching home*, *she could not get money*. *It was difficult and she got into more pain*. *Her relatives decided to take her to the district hospital*. *In discussion with the doctor they decided to hospitalize her for pain management*. *It happened that she could not get money to come back here and the condition got worse until she died*….. *So this situation is very difficult for those who are in the villages*.” (man, group 4). The men, especially those with oesophageal cancer expressed that those treated at Muhimbili National Hospital, for example for surgery would need to have a transport e.g. an ambulance back to ORCI. Patients explained having used their own money to pay for these transports.

Medicines should be provided for free in Tanzania, however they often went out of stock and some patients bought medicines from private pharmacies. Very few could afford to buy medicines and one participant said: “*Until now I have failed to go on with the two treatments that have been prescribed to me*; *radiation and chemotherapy*. *Do you know why*? *I don*’*t have money to buy*. *I took my prescription to my uncle for him to buy me the medications but he returned it to me because he has no money*. *Therefore*, *until now I am only using radiation without chemotherapy*, *because I cannot afford to buy them*.” (woman, group 1).

Patients expressed a need of financial support to pay their children’s school fees. One woman said: “*I am requesting that if possible I should be supported with some money to enable my children to go to school*, *because my children are at secondary school*; *I have no father to my children*, *I am alone*. *I am begging for support to enable my children to go to school*. *For now I am not wealthy as I am not cultivating*, *so I am crying for my children first*.” (woman, group 1).

#### Needs of closeness to cancer care and treatment services

A need of closeness to cancer care and treatment services was expressed. Patients wished to have centres with a capacity to diagnose and treat cancer close to their homes and expressed that their disease, due to delay in diagnosis, had progressed unnecessarily. They had visited dispensaries where the disease had been misdiagnosed and hence mistreated. It was suggested that clinicians in the dispensaries and district hospitals should receive special training to diagnose cancer. One patient said: “*If those in the dispensaries were able to investigate these diseases*, *I think it could have been discovered earlier that I have cancer*. *I have lost a lot of money*; *I am being told it is not the one*. *Now*, *I am requesting the government to make sure that these health care workers get education about cancer diseases or that hospitals where cancer can be treated are opened in many upcountry regions like Mwanza and elsewhere*. *There can be at least three or four other regions for investigation and treatment of cancer diseases*. *The available hospitals in the regions do not manage because there are no specific clinics for cancer*. *For that case this disease becomes a great challenge*, *it is not until the disease has progressed too much that we wake up and think about it*. *When the government discovers it*, *it finds that there are so many that already are affected that it will have a great effect on the economy*.” (man, group 3).

It was mentioned that the hospital capacity at ORCI is low, that the patients are too many compared to the ability of the hospital, and that services are provided at under quality. It was mentioned that up to three patients can use one bed. A man in group 3 said: “*Sometimes they decide to take the patient to the traditional healer*, *and at the traditional healer these diseases are not treated*. *For the case of the Government I would request it to build more hospitals*, *at least one in each region*, *there are many patients*. *This one alone cannot be enough*, *there are so many patients*. *For example it was decided for me to come here in June 16*^*th*^*but I came here in July 31*^*st*^*2007*. *In the whole month of June*, *I was looking for money for transport*. *The Government should try to transport patients who have got referral to come here in order to prevent the disease from being severe*. *And also*, *if expanding the service of the hospitals by building more hospitals*, *it will reduce the congestion of the patients in the wards so that we don*’*t sleep three of us in one bed instead of one*. *As three patients sleep in one bed there is a possibility of cross infection because of contact*.”

A need of information e.g. about the disease, how to live with it, what services to expect, the kind of treatment that should be received, the expected length of stay at the hospital, what nursing care that could be expected, and if there were any privileges provided by any sponsors or government that could be expected, for example money to cover costs for transport back home after treatment was expressed. A wish to be provided with this information in written was expressed. It was also mentioned that some options that in fact were available were not made open.

## Discussion

This study is the first report of HRQOL and needs of care and support of Tanzanians with cancer. A mixed-methods design including quantitative and qualitative research methodology was used. For this purpose the, by our group, newly developed Kiswahili version of the well established EORTC QLQ-C30 was used. The participants, women and men reported a low level of social, role, and physical function and overall health status and quality of life. In spite of this they reported a relatively high level of emotional function. They reported a high level of symptoms, especially pain but also fatigue and insomnia. Financial difficulties were reported to a remarkably high extent by both women and men. The patients reported a number of needs such as need of food and water, hygienic needs, emotional needs, spiritual needs, financial needs, and needs of closeness to cancer care and treatment services. The experiences described in the focus group interviews correspond well with the results obtained with the EORTC QLQ-C30. Furthermore, the needs that were reported in the group interviews are in line with patients’ needs of support described by informal and professional carers in sub-Saharan Africa [7]. Almost no data regarding quality of life exist from Tanzania and comparing the results to other populations is uncertain. Two publications reporting on results using the EORTC QLQ-C30 among cancer patients have been performed for Sub-Saharan Africa. One of these report on a study performed at Kenyatta Hospital in Kenya in which the HRQOL of women with inoperable cervical cancer receiving radiotherapy was investigated [20]. No mean values for scales were presented but the authors conclude that disruption was detected in most domains. A Nigerian study investigating women receiving radiotherapy for breast cancer assessed quality of life with the EORTC QLQ-C30 and reported results in line with the findings presented in this study [21]. The Nigerian study reported higher scores for emotional function and lower scores for role and social function, the highest symptom scores were shown for fatigue, pain, and financial difficulties. Selman et al. [22] investigated HRQOL among patients in palliative care in South Africa and Uganda using the Missoula Vitas Quality of Life Index. It was concluded that patients with cancer reported a better HRQOL compared to patients infected with HIV and that the study sample reported poorer HRQOL compared to similar populations in the US.

Comparing the results from this study to findings from a context in high income countries is challenging as the resources, the care as well as the socio demographic situation differs. Having this in mind, a visual inspection of the results of this study and those from a study on cancer patients in terminal care in Sweden shows that the sample in this study reports a higher function in some respects whereas more pain and remarkably more financial difficulties than the Swedish sample [23].

Satisfactory convergent validity values were revealed for all scales but cognitive function and one item in the fatigue scale, however convergent validity values were also less than satisfactory for the pain and global health/quality of life subscales. Acceptable Cronbach alpha values were revealed for all but three scales: cognitive function, global health/quality of life, and pain. Looking closer to the two items measuring cognitive function one of them assesses the ability to concentrate by reading magazines and watching television. This item appears inappropriate for Tanzanian cancer patients as some are illiterate and inappropriate for the participants in this study as they did not have access to watching television at ORCI. The translation procedure included interviews with 49 patients regarding reactions and thoughts regarding each item in the EORTC QLQ-C30. This particular item revealed difficulties understanding the word “concentrate” but only one patient remarked on the lack of resources to be able to buy magazines. It may be so that the difficulty understanding the word “concentrate” drew attention from the rest of the content in the item. The cognitive function scale showed remarkably weak internal consistency and is recommended to undergo further cultural adaptation. Issues related to culturally dependent activities have been described as potential problems when translating from European to non-European languages [24]. Regarding the global health/quality of life scale, the concepts quality of life and HRQOL are not used in the same way in the East African context and Kiswahili language as in the western world and languages. This scale is also recommended to undergo continued evaluation to fit with the Tanzanian context. The reason for the sub-optimal convergent validity of the pain scale may reflect a cultural difference in expression of pain. Still, the high level of self-reported pain underscores the importance of the scale and the need to assess pain, also highlighted in the focus group interviews.

Approximately 30% of the participants were treated with chemotherapy, whereas approximately 70% with radiotherapy. These treatments are associated with a number of complications including nausea and vomiting, diarrhoea, loss of appetite and weight, fatigue, sleep disturbances, pain, and emotional distress [25]. An advanced disease stage is probably the factor having the greatest impact on the low levels of function and high level of symptoms revealed in this study. Unfortunately the study procedure did not include an evaluation of disease stage. Previous findings [6] from a study investigating referral delay from the rural sector and its influence on the management of patients admitted at ORCI show that of fifty-nine participants 88% were in stage two to four of the disease and approximately 50% in stage three to four.

The high score for pain points out that ORCI is facing severe challenges regarding pain management. Pain management does not require extensive resources and is one of the issues on the agenda of the comprehensive approach to cancer control guided by WHO [26]. Supporting previous research the findings of this study underscore the importance of relieving patients from cancer-related pain [27]. Oral morphine, reconstituted from powder is available at ORCI, however, to what extent is uncertain. One may expect that availability differs over time. Looking at the rest of East Africa Uganda stands out as a good example with regard to supportive care, being the first country in Africa to provide palliative care for people with HIV and cancer, a priority in its National Health Plan for 2001 to 2005 [28].

The participants reported financial difficulties to a high extent with the EORTC QLQ-C30 and in the focus-group interviews. Tanzania is one of the poorest countries in the world [29]. The participants came from economically very poor groups such as peasants, small scale business people, low level cadre employees, and non-employed. The cancer disease caused extra costs e.g. for transports, medicines, food, and water. Some had to travel very far to reach ORCI and due to treatment but also lack of money had to spend months even a year at the hospital which in turn caused feelings of loneliness, insecurity, and isolation. In spite of reporting a relatively high emotional function with the EORTC QLQ-C30 participants expressed a need of emotional care in the focus-group interviews. The findings indicate that the patients at ORCI, as shown for cancer patients in many contexts e.g. in Bahrain, would benefit emotionally if visited by relatives or support groups [30]. Also in low income countries governments are responsible for the wellbeing of their citizens. Unfortunately, often they don’t have at their disposal the necessary resources to provide the required psychosocial care for somatic patients.

A reliance on both traditional and allopathic medical systems often results in late presentation. There are only three hospitals in Tanzania where cancer can be diagnosed, and patients from all over the country have to travel to ORCI in Dar es Salaam for cancer treatment. Biopsies are sometimes taken at another centre and it can take up to six months to get a result from these. The capacity to examine biopsies is around 2,000 specimens per year, but over 10,000 samples are received. Specimens sent to Europe are often diagnosed in three weeks. A biopsy can be ‘fast tracked’ at ORCI within two weeks but costs the equivalent of US$ 25 which many patients are unable to afford.

Several aspects for which a need was mentioned in the focus group interviews are not included in the EORTC QLQ-C30 such as food, water, and hygiene. Both men and women expressed a need for food and water. During the study period patients at ORCI were provided with food for free. However patients expressed special needs due to their disease and subsequent treatment which were not met. Water was not provided for free and patients expressed a need of bottled water. Patients had access to tap water which is not safe for drinking unless boiled.

Relatively few men participated in this research and a visual inspection of the EORTC QLQ-C30 data does not reveal any considerable differences between women and men. However, women reported a somewhat better HRQOL than men, a different pattern from that found in western countries where women tend to report more distress than men. This illustrates a potential cultural difference of interest to illuminate in future research. When considering the findings of this research it should be considered that the sample is relatively small, probably biased towards inclusion of persons willing to share their concerns and in stage two or more of the disease which could hamper the representativeness of the findings. In spite of this we consider the findings as representative for the patient population seen at ORCI at the time of our study. The fact that almost all cancer patients in Tanzania receiving treatment for their cancer disease are seen at ORCI supports the possibility to generalize the findings to the population of Tanzanian cancer patients.

In Tanzania the government, parastatal organizations, voluntary organizations, religious organizations, private practitioners, and traditional medicine provide health services [31]. The Government’s referral system has a pyramidal approach starting from dispensary to hospitals. Most often a cancer diagnosis relies on simple and cost effective technology, however cancer care and treatment is dependent on trained and skilled health care workers, not available at the lower level health facilities [32]. The findings from the focus-group interviews show that patients had visited the lower level facilities without being properly diagnosed.

## Conclusions

Cancer patients in Tanzania seen at Ocean Road Cancer Institute in Dar es Salaam report a low level of health-related quality of life, a high level of symptoms, and a large number of unmet needs of e.g. water and food, hygiene articles, emotional and financial support, pain relief, and access to the health care. The high score for pain points out that ORCI is facing severe challenges regarding care and treatment. However, when considering this finding it should be noted that the pain subscale of the Kiswahili version of the EORTC QLQ-C30 did not reach acceptable internal consistency and showed less than satisfactory convergent validity. This also applies to the subscales cognitive function and global health/quality of life. Attention should be drawn to meet the identified needs of Tanzanian cancer patients while hospitalized but also when at home. Increased accessibility of mosquito nets, pads, and pain-killers would help to fulfil some needs.

## Abbreviations

HRQOL: Health-Related Quality of Life; ORCI: Ocean Road Cancer Institute; QLQ-C30: Quality of Life Questionnaire (Version 3); IAEA: International Atomic Energy Agency; PACT: Program of Action for Cancer Therapy; WHO: World Health Organization; RN: Registered Nurse.

## Competing interests

The authors declare that they have no competing interests. There are no financial and/or personal relationships between the authors and others that might bias the work.

## Authors’ contributions

**GMM** – Participated in the procedure to translate the EORTC QLQ C-30 into Kiswahili, completed ethical clearance, collected qualitative and quantitative data, analyzed qualitative data, drafted the manuscript, and approved the final version of the manuscript for publication. **LW** – Planned and participated in the procedure to translate the EORTC QLQ-C30 into Kiswahili, developed the research plan and design, initiated and completed ethical clearance, analyzed quantitative data, drafted the manuscript, and approved the final version of the manuscript for publication. **TWK** – Participated in the procedure to translate the EORTC QLQ C-30 into Kiswahili, drafted the manuscript, and approved the final version of manuscript for publication. **LvE** - Planned and participated in the procedure to translate the EORTC QLQ-C30 into Kiswahili, developed the research plan and design, initiated and completed ethical clearance, analyzed quantitative and qualitative data, drafted the manuscript, and approved the final version of the manuscript for publication.
